# Comparing Anesthesiology Residency Training Structure and Requirements in Seven Different Countries on Three Continents

**DOI:** 10.7759/cureus.1060

**Published:** 2017-02-26

**Authors:** Satoshi Yamamoto, Pedro Tanaka, Matias V Madsen, Alex Macario

**Affiliations:** 1 Department of Anesthesiology, University of Texas Medial Branch at Galveston; 2 Department of Anesthesiology, Perioperative and Pain Medicine, Stanford University School of Medicine; 3 Department of Anesthesiology, Herlev and Gentofte Hospital, University of Copenhagen

**Keywords:** anesthesia, residency, residency structure, graduate medical education, in the world

## Abstract

Little has been published comparing the graduate medical education training structure and requirements across multiple countries. The goal of this study was to summarize and compare the characteristics of anesthesiology training programs in the USA, UK, Canada, Japan, Brazil, Denmark, and Switzerland as a way to better understand efforts to train anesthesiologists in different countries.

Two physicians trained in each of the seven countries (convenience sample) were interviewed using a semi-structured approach. The interview was facilitated by use of a predetermined questionnaire that included, for example, the duration of post-medical school training and national requirements for certain rotations, a number of cases, faculty supervision, national in-training written exams, and duty hour limits. These data were augmented by review of each country’s publicly available residency training documents as available on the internet.

Post-medical school anesthesia residency duration varied: three years (Brazil), four years (USA), five years (Canada and Switzerland), six years (Japan and Denmark) to nine years (UK), as did the number of explicitly required clinical rotations of a defined duration: zero (Denmark), one (Switzerland and UK), four (Brazil), six (Canada), and 12 (USA). Minimum case requirements exist in the USA, Japan, and Brazil, but not in the other countries. National written exams taken during training exist for all countries studied except Japan and Denmark. The countries studied increasingly aim to have competency-based education with milestone assessments. Training duty hour limits also varied including for example 37 hours/week averaged over a one month with limitations on night duties (Denmark), a weekly average of 48 hours taken over a 17 week period (UK), 50 hours/week maximum (Switzerland), 60 hours/week maximum (Brazil), and 80 hours/week averaged over four weeks (USA).

Some countries have highly structured training programs with multiple national requirements with training principally carried out at a home institution. Other countries have a more decentralized and unregulated approach with fewer (if any) specific case or rotation requirements, where the trainee creates his/her own customized training to meet broad objectives and goals. The countries studied have different national training requirements, unique duty hour rules and are at varying stages in transitioning to an outcome based model of residency.

## Introduction

Although some descriptions of the methodology of training in anesthesia in individual countries have been published [1], few if any comparative analyses exist comparing the graduate medical education structure and requirements across multiple countries for any of the medical specialties.

This is at a time when regulatory bodies in different countries are changing training requirements. For example, the Next Accreditation System was launched in the USA by the Accreditation Council for Graduate Medical Education (ACGME) in 2012 as a shift towards competency-based medical education where educational outcomes are emphasized instead of the traditional structure and process based approach of graduate medical education [2]. In Europe, ongoing efforts exist to develop appropriate minimum standards of quality, content, and duration of training for all European countries [3]. New training guidelines proposed by the European Board and Section of Anaesthesiology offer an outcomes-based curriculum and aim to reduce differences in training among countries. The first anesthesiology training guidelines as part of the Charter on Training of Medical Specialists were published in 1996. These were updated in 2012 to include 10 domains of general core competencies and seven domains of specific core competencies [4].

The goal of this study was to summarize and compare the characteristics of anesthesiology training programs in the USA, UK, Canada, Japan, Brazil, Denmark, and Switzerland as a way to better understand efforts to train anesthesiologists in different countries.

## Materials and methods

After human subjects approval, two physicians trained in each of seven countries (convenience sample) were interviewed by the first author using a semi-structured approach facilitated by the use of a predetermined questionnaire about national requirements of anesthesia training programs in each respondent’s home country.

Specific items of interest included: duration of post-medical school training, minimum case number or rotation requirements, use of competency-based curriculum, faculty supervision, duty hour limits and national in-training written exams.

Questionnaire data were augmented by review of each country’s publicly available residency training documents as available on the Web and by visiting the official website of the governing anesthesia body in each country.

## Results

Selection of applicants for anesthesia training differs between the countries studied. A mathematical computer algorithm is used to match an applicant to the program most preferred on that applicant’s rank order list in the USA and Canada. Other countries have candidates directly apply to a particular region or facility for training (Table 1).

Years of training after medical school ranged from three (Brazil), four (USA), five (Canada and Switzerland), six (Japan and Denmark) to nine (UK).

Direct faculty supervision of residents was required in all countries at all times except under some circumstances in the UK and Denmark (Table 2).

Requirements for a minimum number of certain case types exist in the USA, Japan, and Denmark but not in the other countries (Table 3 and 4).

Training duty hour limits also varied for example 37 hours/week averaged over a one month with limitations on night duties (Denmark) to 80 hrs per week (USA) (Table 5).

**Table 1:** Residency program application process and training duration for each country 1. A mathematical computer algorithm to match an applicant to the program most preferred on that applicant’s rank order list.http://www.nrmp.org/match-process/match-algorithm/ 2. The algorithm compares applicant and program rank order lists and matches applicants to programs based on both partie's stated preferences 3. http://www.carms.ca/en/residency/match-algorithm/ http://www.rcoa.ac.uk/training-and-the-training-programme/the-stages-of-training

	USA	UK	Canada	Japan	Brazil	Denmark	Switzerland
Application process	Match^1^	National application process for postgraduate foundation years one-two and then assigned deanery and hospital, then another similar national application for core and specialist anesthesia training	Match^2^	Match for the first two years and application to institution for four-year anesthesia residency	Application to institution	Lottery pick for postgraduate year, application to separate institutions for postgraduate year 2, & then application for anesthesia residency in one of three regions in country	No specific residency programs. candidates put together own curriculum & apply for rotations at different teaching hospitals & make sure to meet all requirements
Written exam required to be admitted to residency	N	N	N	N	Y	N	N
Post medical school training duration (years)	one (internship) + three (anesthesia)	two (foundation years) + two (core anaesthesia) + two (intermediate) + three (higher (+/- advanced).^3^	1.5 (general medicine) + 3.5 (anesthesia)	two (general medicine) + four (anesthesia)	zero + three (anesthesia)	one (general medicine) + one (introductory anesthesia year) + four (anesthesia)	Minimum of five years
Total years post medical school	four	nine	five	six	three	six	five

 

**Table 2:** Overview of residency program structure 1. Voluntary, self-created elective experience can be proposed to program 2. http://acgme.org/acgmeweb/Portals/0/PDFs/Milestones/AnesthesiologyMilestones.pdf 3. Work Place Based Assessments as per http://www.rcoa.ac.uk/training-anaesthesia/rules-and-regulations-governing-training-%E2%80%93-the-curriculum 4. http://www.dasaim.dk/wp-content/uploads/2014/01/Curriculum-for-Specialist-Training-in-Anaesthesiology-Core-Training-Programme.pdf 5. Every trainee must, at all times, be responsible to a nominated consultant. The consultant must be available to advise and assist the trainee as appropriate. Sometimes this will require the consultant’s immediate presence but on many occasions less direct involvement will be needed. Indirect supervision falls into three categories: local, distant and remote sites. Local supervision: The supervisor is usually within the theatre suite immediately available for advice and is able to be with the trainee within minutes of being called. Distant supervision: The supervisor is available rapidly for advice but is off the hospital site and/or separated from the trainee by over 10 minutes. Supervision in remote sites: any location in which it cannot be guaranteed that the help of another anaesthetist will be available. This may be either within or away from the base hospital (http://www.rcoa.ac.uk/system/files/TRG-CU-CCT-ANAES2010.pdf) 6. As more milestones achieved more autonomy allowed not requiring direct supervision 7. A non-mandatory in training test given by the Japan Organization of Advancing Medical Education Program is usually taken during PGYtwo before clinical anesthesia meant to evaluate medical knowledge and skills in general, not specific to anesthesia 8. The American Board of Anesthesiology offers the BASIC Examination at the end of the second postgraduate year, and a passing score is required to graduate residency. 9. While a resident may graduate from a residency program without being successful on their board exams (certification exams are written in the last year of training), they will not be conferred their (Fellow of The Royal College of Physicians of Canada) FRCPC designation, and thus they cannot work independently as an anesthesiologist in a major center 10. Includes the Basic written exam after postgraduate year two, the advanced written exam after finishing residency, and the applied exam thereafter which includes oral exams, and beginning in 2018 OSCEs (http://www.theaba.org/Exams/APPLIED-(Staged-Exam)/About-APPLIED-(Staged-Exam) 11. http://www.rcoa.ac.uk/training-and-the-training-programme/the-stages-of-training 12. Includes written, interview, and skill tests 13. Switzerland uses the Part I of the European Society of Anesthesiology European Diploma in Anaesthesiology and Intensive Care written exam, followed by a Swiss specific oral Exam 14. Board certification requirements are changing with the new system taking place in 2019

	USA	UK	Canada	Japan	Brazil	Denmark	Switzerland
Electives (non-required clinical rotations available)	Y	Y	Y	Y	Y	Y^1^	Y
Competency Based Curriculum	Y	Y	Y	Y	N	Y	Y
Milestones Based Assessment	Y^2^	Y^3^	N	N	N	Y^4^	Y
Direct Faculty Supervision Required for All Years of Training	Y	N^5^	Y	Y	Y	N^6^	Y
National In-Training Written Exams	Y	Y	Y	N^7^	Y	N	Y
Need to Pass Exam to Graduate Residency	Y^8^	Y	N^9^	N	N	N	N
Board Certification Test	Y^10^	Y^11^ (Fellowship of the Royal College of Anaesthetists; after postgraduate year six)	Y	Y^12^	Y	N	Y^13^
Name of Residency Accreditation Organization	Academic Council of Graduate Medical Education	General Medical Council, Royal College of Anaesthetists	Royal College of Physicians and Surgeons of Canada	Japanese Medical Specially Board^14^	Education Administration of Brazilian Society	National Board of Health	Swiss Society of Anesthesiology

 

**Table 3:** Overview of rotation and case requirements Y=Yes, N=No a. http://www.rcoa.ac.uk/training-and-the-training-programme/the-stages-of-training b. Rotations with fixed duration are not required but nine general competencies (disease management, patient assessment and preoperative preparation, intraoperative care, postoperative patient care and pain management, resuscitation and emergency management, practical anesthetic procedures/skills, quality management and health economics, anesthesia non-technical skills, professionalism and ethics, and education, self-directed learning, research) and the eight specialty types listed in Table 2. c. ACGME minimum required number of cases and procedures (https://www.acgme.org/Portals/0/PFAssets/ProgramRequirements/040_anesthesiology_2016.pdf) Pediatrics (< three months old) = five, Pediatrics (< three years old) = 20, Pediatrics (< 12 years old) = 100, Spinal = 40, Epidural=40, Peripheral Nerve Block = 40, Life-Threatening Pathology = 20, Cardiac = 20, Intrathoracic Non-Cardiac = 20, Vascular Major Vessels = 20, Vaginal Delivery = 40, Cesarean Section = 20, Pain Evaluation – New Patient = 20, Intracerebral = 20, Intracerebral Open = 11. d. Pediatric (< six yrs), 25 cases; cesareans, 10 cases; cardiovascular, 25 cases; thoracic, 25 cases; neurosurgery, 25 cases e. According to regulation of education and training centers -2016- Brazilian Society of Anesthesiologists: I- Pre and postoperative: minimum of 10% of the annual workload for pre-anesthetic evaluation (preoperative assessment clinic and pre-anesthetic evaluation), post-anesthetic evaluation, treatment of postoperative pain and acute and chronic pain syndromes;II - Intensive care unit and emergency department: minimum of 15% of the annual workload; III- Elective anesthesia, diagnostic and therapeutic services: minimum of 45% of the annual workload; IV-obstetrics: minimum of 10% of the annual workload; V-elective rotations: cardiology, pulmonology, neurology, clinical pathology laboratory, physiology lab, pharmacology laboratory, experimental surgery and transfusion medicine or others at the discretion of the institution. It is also mandatory anesthetic procedures for general surgery, obstetrics and children age between zero-12 years and also to do at least three of the following surgical specialties: proctology, vascular surgery peripheral, orthopedics and traumatology, gynecology, otolaryngology, ophthalmology, urology, diagnostic tests, thoracic-lung surgery and neurosurgery. Provide a minimum of 440 anesthetic acts or 900 annual hours of practical training in anesthesia for each resident, covering mandatorily anesthetic procedures for general surgery, obstetrics and children zero-12 years and also to at least three of the following specialties surgical: proctology, peripheral vascular surgery, orthopedics and traumatology, gynecology, otolaryngology, ophthalmology, urology, diagnostic tests, thoracic-lung surgery and neurosurgery

	USA	UK	Canada	Japan	Brazil	Denmark	Switzerland
Rotation requirements	Y	Y^a^	Y	N	Y	Y	Y^b^
Case Number Requirements	Y^c^	N	N	Y^d^	Y^e^	N	N

**Table 4:** Rotation requirements with specified duration in months (if cell in Table is empty no requirement exists) *The UK has ‘core specialty’ requirements, but apart from ICU do not need to be accomplished in dedicated blocks ** Rotation periods are not clearly specified. To match milestone based targets, many resident programs provide rotation periods for OB, Ped, Cardiac, Neuro and Thoracic ***no duration specified as milestones based # These are “specific core competencies” of the Swiss catalogue of objectives in anesthesia and reanimation. No duration is specified. In smaller hospitals the experience may not be separate rotations as residents perform samples cases in each of the domains. In bigger hospitals such as University Hospitals, cardiac anesthesia, pediatric anesthesia, neuroanesthesia, prehospital emergency, and pain service may be separate rotations

	USA	UK*	Canada	Japan**	Brazil	Denmark***	Switzerland^#^
OB	two	Yes	two		one	Yes	Yes
Pediatrics	two	Yes	three		one	Yes	Yes
Cardiac	two	Yes			one		Yes
Neuro	two	Yes					Yes
Pain Service	three	Yes			one	Yes	
ICU	four	nine	three			Yes	six
Acute Pain	one	Yes					
Chronic Pain	one	Yes	one				Yes
Regional	one	Yes					
Pre-op	0.5						
PACU	0.5						
General Anesthesia		Yes	12				
Adult Internal Medicine		Yes	six				
Thoracic		Yes				Yes	Yes
ENT		Yes				Yes	Yes (Airway surgery/management)
Ophthalmic						Yes	
Vascular		Yes				Yes	
Orthopedic		Yes				Yes	
Trauma		Yes				Yes	
Emergency Medicine						Yes	
Anesthesia outside OR	0.5	Yes				Yes	Yes
Abdominal surgery		Yes				Yes	
Ambulatory		Yes				Yes	
Gynecologic		Yes				Yes	

 

**Table 5:** Duty hour limit requirements ^http://www.rcoa.ac.uk/careers-and-training/working-time-regulations ^^The Labor Standards Act applies for all workers in general and limits are eight hours a day, 40 hours a week in total. Many medical residencies may not be compliant. Resident overtime is controlled by the agreement for overtime work regulation: overtime is allowed within 45 hours per month, 360 hours per year. ^^^In some departments, trainees may work more than 37 hours, but the extra hours are either paid times one and a half or they will get the time off in the next period.

USA	UK	Canada	Japan	Brazil	Denmark	Switzerland
80 hr/week, averaged over four weeks; one day off/week; 10 hours off between shifts	Weekly average of 48 hours taken over a 17 week period^	24 hours for a single call shift and no more than seven call shifts every 28 days. Varies from province to province. In Alberta, call shifts can be up to 26 hours. Call shifts are limited to 16 hours in Quebec.	Yes^^	60 hours/week	37 hours/week averaged over a one month (national labor law for all employees), with limitations on night duties^^^	50 hrs per week maximum

## Discussion

Some countries offer highly structured training programs with multiple national requirements and training principally provided at one primary facility, whereas other countries have a more decentralized and unregulated approach with fewer if any, case or rotation requirements.

Different countries are at various stages in reaching an outcome based model of residency. In Canada the competence by design initiative will transition graduate medical education from a traditional time-based model to a hybrid form of competency-based medical education [5]. Denmark already has specific entrustable professional activities that allow assessment of residents as they progress through their training. These entrustable professional activities are a method to translate competencies (descriptors of physicians) into descriptors for clinical practice [6]. As a European comparator, Switzerland has a common competency-based curriculum (the Swiss catalogue of objectives in anesthesia and reanimation [7]) and aims to have entrustable professional activities based curriculum within the next 10 yrs.

Japan implemented major revisions to residency requirements and anesthesia board qualification system in 2015. Historically in Japan, to address the need for anesthetists an “anesthesia provider “qualification existed requiring a minimum of 300 cases or two years of anesthesia training. However, since 2015, all Japanese medical practitioners are now required to graduate a two year primary residency with rotations for many departments including surgical, internal and ambulatory medicine. After this two-year primary training, residents are eligible to apply for the four-year anesthesia residency, and when completed can then apply for board certification.

The varied structure of training programs and board certification processes between countries may create challenges for national medical boards to assure quality of training among anesthesiologists that wish to practice in countries outside the one they trained. Many European countries have adopted written and oral board exams administered by the European Society of Anesthesia [8].

The design and components of the optimal training method of anesthesiologists are unknown. The differences in training among residency programs even within the same continent may be in part due to different functions that anesthesiologists are expected to serve in each country. For example, compared to the USA the scope of practice for anesthesiologists in Western Europe may include prehospital emergency medicine that encompasses more complex front-line decision-making and requires specific residency training [9]. Similar to some medical centers in the USA with the advent of the perioperative surgical home, European anesthesiologists are actively engaged not only in the intensive care unit (ICU) and operation room management, as well as in anesthesia preoperative assessment centers and pain clinics.

A barrier in creating international minimum training standards, is that differences exist in taxonomy of current competencies. For example, the ACGME in the USA outlines six core competencies (patient care, medical knowledge, interpersonal skills and communication, systems-based practice, professionalism, and practice-based learning and improvement) whereas the Canadian Medical Education Directives for Specialists utilizes different terms (medical expert, scholar, communicator and collaborator, leader, professional, and health advocate). Switzerland, as an example of how European countries differ from North America, specifies its own set of ten core competencies [i].

Work hours also vary between different countries. As a result of the European working time directive 2003/88/EC, many European countries were legally mandated to decrease their weekly working hours to 58 hours in 1998, then 56 hours in 2007, and to a maximum of 48 a week in 2009 [10-11]. Other regulations exist such as the longest continuous shift of 13 hours with an obligatory 11 consecutive hours of rest per 24 hour period, a rest period for every six hours worked, weekly rest (at least 24 hours uninterrupted) and annual leave (at least four weeks). One large survey study, however, found that less than 40% of European neurosurgical trainees actually comply with the 48-hr week limit [12].

Duty hours restrictions have also been the subject of scrutiny in the USA. In 2011, the ACGME first limited trainee work hours to no more than 80 hours per week. The impact of this change on education and practical training still remains to be elucidated. Some studies suggest that work hours restrictions may have had the unintended consequence of adversely impacting patient care thought to be due to a lack of continuity of care and increased the frequency of handoffs [13-14].

Only the USA, Canada, and Brazil specify that certain rotations be a required minimum duration. Trainees in the UK, in contrast, for example, spend three to six months at a time rotating at different hospitals in the geographical region (of the deanery) and during that time the trainee is expected to have their clinical experience units and knowledge and competencies be signed off. At annual review, overall progression is assessed by deanery-appointed consultants, but at each hospital, there is one consultant who is designated as the ‘college tutor’ for residents to consult, with regards to their progression.

With regard to clinical training, case logs may be utilized as a surrogate for clinical competency. Specified national requirements for a minimum number of cases in various subspecialties currently exist in the USA. Other countries such as Brazil (minimum of 440 anesthetics or 900 annual hours) have overall case minimums but do not break it down further into actual subspecialty case numbers. Although Brazil has no specific minimum number of certain subspecialty case types, there are minimum annual workload percentages for the following domains: pre and postoperative care (minimum of 10% of the annual workload), ICU and emergency department (minimum of 15%), elective anesthesia (minimum of 45% of the annual workload) and obstetrics (minimum of 10%) [15].

However, despite specified national requirements within some countries, a study at one USA training program demonstrated even though every resident exceeded the minimums, there is two-three fold variability in the number of cases that residents will participate in within the same residency [16]. Furthermore, although case minimums provide a best-guess estimate of the number of cases an average trainee needs, they do not account for outliers at either end of the learning spectrum who may learn faster or slower. As the movement toward competency and outcomes based training moves forward, regulatory bodies may choose to eliminate the specific case number requirements.

Methodologies (e.g., use of a written exam as criteria) used in different countries to select medical graduates for residency positions also varies [17]. The actual mechanism by which applicants end up in a particular anesthesiology residency varies greatly across the seven countries studied. Canada and the USA, for example, employ a national resident match program that links candidates with residencies via a computer algorithm. In contrast, medical students in the UK apply through a national application process, where students rank deaneries (regional organizations) in order of preference. Applicants are scored according to a number of selection criteria (educational achievements score and a situational judgment test) and then assigned to a deanery (and subsequently hospitals within the region) for foundation years one and two. There are additional similar national application processes for core and specialist anesthesia training once the foundation years are completed. Denmark uses a lottery pick for the first post-graduate year with subsequent application needed to separate institutions for the second post-graduate year and thereafter apply for anesthesia residency in one of three regions within the country.

Limitations

The questionnaire items used to collect the data for this study from the participating physicians that had trained in each country presumed a training system like the one in the USA. However, in some countries (Switzerland) there are no specific residency programs. Instead, candidates construct they own curriculum, apply for rotations at different teaching hospitals and assume responsibility for meeting all the required criteria when applying for board-certification.

There was interest initially in asking about any existing classroom-based didactics in the training in each country. However, the type and amount of formal teaching sessions outside the clinical setting anecdotally vary a lot among institutions within the same country so comparing those activities across countries didn’t yield useful data. Further studies are warranted to assess this issue.

This study also did not assess specialization after residency such as availability of fellowships in clinical sub-specialties like pediatric anesthesia for example. The study also did not assess staffing arrangements among countries. Some countries mandate two professionals to provide anesthesia care in the operating room while others may have varying roles and supervision for physician assistants or nurse anesthetists [18]. Other characteristics such as the use of simulation, either for teaching or assessment [19], or the use of formal teaching sessions [20], or the attention paid to faculty development in education [21] in each of the countries were not specifically determined.

[i] Disease management, patient assessment and preoperative preparation, intraoperative care, postoperative patient care and pain management, resuscitation and emergency management, practical anesthetic procedures/skills, quality management and health economics, anesthesia non-technical skills, professionalism and ethics, and education, self-directed learning and research.

## Conclusions

Although apprenticeship remains the primary way of teaching overall, each country has unique training structure and characteristics. Many countries have different national training requirements, unique duty hour rules and are at varying stages in transitioning to an outcome based model of residency. Some countries have a structured education within a dedicated network of training hospitals in which the resident completes all necessary training. Other countries have a less structured approach, where the trainee creates customized training rotations that meet broad objectives and goals. These differences in training requirements, program design and board-certification processes between countries remain of unclear significance with regard to how they affect clinical care and whether countries can improve their training programs based on what is being done in other countries.
